# Subgenomic RNA Detection in SARS-CoV-2 Assessing Replication and Inactivation Through Serial Passages, RT-qPCR, and Electron Microscopy

**DOI:** 10.3390/ijms26031281

**Published:** 2025-02-01

**Authors:** Talita da Silva França, Juliana Fernandes Amorim da Silva, Gabriella Christine Neves da Silva, Barbara Oliveira dos Santos, Stephanie Almeida Silva, José Henrique Resende Linhares, Marcos Alexandre Nunes da Silva, Debora Ferreira Barreto-Vieira, Vanessa Salete de Paula, Liliane Monteiro de Morais, Renata Tourinho Santos, Gisela Freitas Trindade

**Affiliations:** 1 Virological Technology Laboratory, Bio-Manguinhos/FIOCRUZ, Rio de Janeiro 21040-900, Brazil; talita.franca@bio.fiocruz.br (T.d.S.F.); barbara.santos@bio.fiocruz.br (B.O.d.S.); stephanie.silva@bio.fiocruz.br (S.A.S.); jose.henrique@bio.fiocruz.br (J.H.R.L.); liliane@bio.fiocruz.br (L.M.d.M.); renata.tourinho@bio.fiocruz.br (R.T.S.); 2 Immunomolecular Analysis Laboratory, Bio-Manguinhos/FIOCRUZ, Rio de Janeiro 21040-900, Brazil; juliana.silva@bio.fiocruz.br (J.F.A.d.S.); gabriella.silva@bio.fiocruz.br (G.C.N.d.S.); 3 Viral Morphology and Morphogenesis Laboratory, Oswaldo Cruz Institute/FIOCRUZ, Rio de Janeiro 21040-900, Brazil; marquinhosans@gmail.com (M.A.N.d.S.); barreto@ioc.fiocruz.br (D.F.B.-V.); 4 Molecular Virology and Parasitology Laboratory, Oswaldo Cruz Institute/FIOCRUZ, Rio de Janeiro 21040-900, Brazil; vdepaula@ioc.fiocruz.br

**Keywords:** SARS-CoV-2, sgRNA, RT-qPCR

## Abstract

Subgenomic RNAs (sgRNAs) are potential markers of active SARS-CoV-2 replication, serving as templates for the synthesis of structural and accessory proteins in infectious viral particles. This study aimed to use RT-qPCR to quantify sgRNA and negative RNA intermediates, assessing viral replication in virus samples inactivated by β-propiolactone (βPL). Inactivated viruses subjected to five blind serial passages (BSs) were amplified by RT-qPCR using primers to target the envelope (ENV) and nucleoproteins (N1 and N2) of genomic genes, subgenomic envelope RNA (sgENV), and intermediate envelope RNA (ENV-). All positive controls showed consistent viral titers across passages (10 log10 copies/mL in N1/N2 and 11 log10 copies/mL in ENV) during BSs. Inactivated viral samples for ENV and ENV- targets ranged from 11.34 log10 copies/mL in BS1 to 11.20 log10 copies/mL in BS5. The sgENV was no longer detected in the inactivated SARS-CoV-2 samples after the second passage, suggesting successful inactivation. Replication kinetics showed consistent profiles for N1/N2, ENV, and ENV- targets in the first three post-infection hours (pih) and maintained approximately 5 log10 copies/mL at 1 pih, 2 pih, and 3 pih. A sharp exponential increase in the viral titer was observed from 24 pih onwards, peaking at 11.64 log10 copies/mL at 48 pih. Transmission electron microscopy confirmed viral particles only in cells infected with active SARS-CoV-2. These results support the use of sgRNA as a reliable marker for SARS-CoV-2 replication, especially in distinguishing between active replication and non-viable particles and in the development of diagnostic and therapeutic strategies.

## 1. Introduction

SARS-CoV-2 is a pathogenic virus composed of structural proteins, such as Spike (S), Envelope (E), and Membrane (M) proteins, and a positive-sense single-stranded RNA genome approximately 30 kb in length, encapsulated by a nucleocapsid (N). This virus can infect cells through the endocytic pathway by interacting with cellular receptors, such as angiotensin-converting enzyme 2 (ACE2) and cell surface serine protease (TMPRSS2) [1,2]. Copies of the complete genomic RNA (gRNA) are produced and incorporated into new viral particles during replication [1,2,3]. Subgenomic RNAs (sgRNAs), generated through discontinuous transcription [4,5], encode proteins essential for assembling new SARS-CoV-2 particles, such as Spike (S), Envelope (E), and virus accessory proteins [5,6]. Previous studies showed that sgRNAs can be detected shortly after the virus enters the cell and that they are poorly incorporated into mature virions [7,8], which suggests that sgRNA would be indicative of SARS-CoV-2 replication. Using RT-qPCR to quantify sgRNA and intermediate RNAs allows us to assess the virus’s active replication. RT-qPCR for sgRNA can provide important parameters to confirm viral inactivation and viral load in samples infected with SARS-CoV-2 when it is used along with gRNA quantification. Currently, the RT-qPCR methodology is used to detect SARS-CoV-2 gRNA; however, it is not capable of discriminating against the replicating RNA from the RNA of defective particles, or non-replicating RNA [9], which can overestimate the number of viable viral particles. The RT-qPCR technique was adopted to quantify sgRNA and intermediate RNAs, assessing viral replication in β-propiolactone (βPL)-inactivated samples. This promotes viral inactivation through the alkylation of viral RNA, a methodology extensively described in the scientific literature and adapted for use in the production of non-infectious viruses. A study previously conducted by our group indicated that inactivation by βPL is the most effective strategy for viral inactivation due to its minimal impact on the morphology of the viral particle and its capability to induce an immune response in a murine model [10]. Consequently, this methodology can be employed in the development of immunobiological products and in technological development projects.

## 2. Results

### 2.1. Reducing sgRNA Levels in Inactivated SARS-CoV-2 Samples

**Figure 1:**
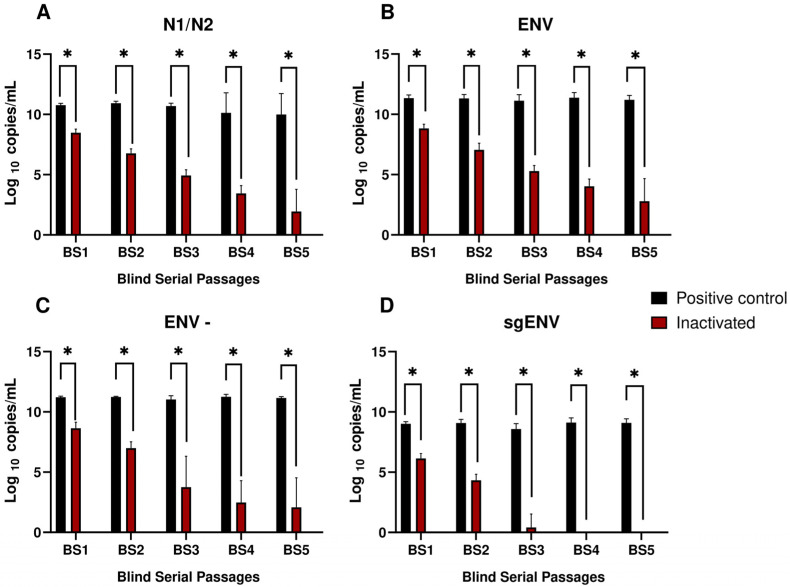
Quantification of SARS-CoV-2 subjected to blind serial passages in Vero E6 Cells using different targets. (**A**) Target N1/N2, (**B**) Target ENV, (**C**) Target ENV-, (**D**) Target sgENV. Black bars indicate positive control quantification, red bars indicate inactivated SARS-CoV-2 quantification, and green bars indicate MOCK quantification throughout blind passages. Mean values obtained for each target were calculated separately to define N1 and N2 target values. BS, blind serial passage; * *p*-value < 0.05.

The gRNA quantification through genomic targets N1/N2 and ENV showed constant values in BS positive control samples. The same profile was observed for ENV- and sgRNA in the same samples. Conversely, samples inactivated with β-propiolactone exhibited a reduced viral load in all targets (Figure 1A–D).

These samples showed at least a 2-log reduction in comparison with the positive control when targets N1 and N2 were quantified (Figure 1A). The mean viral load was 8.48 log10 copies/mL, with a standard deviation (SD) of 0.30, in BS1. This target’s level decreased in subsequent passages and reached 1.94 (SD = 1.83) in BS5. Furthermore, ENV detection in inactivated samples showed a similar profile to N1 and N2 (Figure 1B). A significant decline (*p* < 0.05) in ENV levels was observed between passages 2 and 3, when the mean viral load reached 5.30 (SD = 0.47). Lower viral loads were observed in the final passages, and they reached 2.80 (SD = 1.88) in BS5.

With respect to the ENV- target, inactivated samples showed a mean viral load of 8.65 (SD = 0.49) in BS1 (Figure 1C). A 3-log decline was observed in BS3 and it persisted up to the final passages, at a load of 2.08 (SD = 2.44) in BS5. Regarding sgENV, inactivated samples showed a reduction in viral load to undetectable levels (Figure 1D). The mean viral load in BS1 was 6.28 (SD = 0.40). The sgENV levels decreased between BS1 and BS2. They were quantified at 4.50 (SD = 0.49). This sgENV reduction persisted, and the target became undetectable in BS3. According to the Mann–Whitney test, the positive control and the inactivated samples recorded a *p*-value < 0.05.

### 2.2. SARS-CoV-2 Replication Kinetics

**Figure 2:**
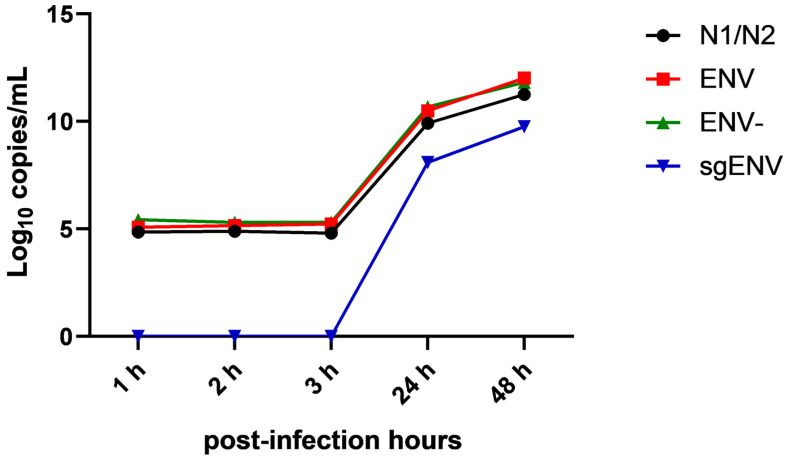
Quantification of N1/N2, ENV, ENV-, and sgENV in SARS-CoV-2 subjected to replication kinetics in Vero E6 cells. The black line points out N1/N2 quantification, the red line highlights ENV quantification, the green line shows ENV- quantification, and the blue line indicates sgENV quantification throughout the replication kinetics.

Samples from replication kinetics were subjected to RT-qPCR analyses to better understand the SARS-CoV-2 replicative profile. Viral loads detected during the initial kinetic stages remained consistent in N1, N2, ENV, and ENV- targets. An exponential increase by 5 logs was observed between 3 pih and 24 pih, and it was followed by an increase close to 2 logs in viral load at the last point (48 pih). Unlike the previous findings, amplification of the sgENV target was not clear during the early kinetic stages (1 pih to 3 pih). However, the viral load was detected at high levels (8.10 log10 copies/mL and 9.76 log10 copies/mL at 24 pih and 48 pih, respectively) from 24 pih onwards. All negative controls employed throughout the RNA extraction and RT-qPCR procedures consistently showed no viral load for the assessed target (Figure 2).

**Figure 3:**
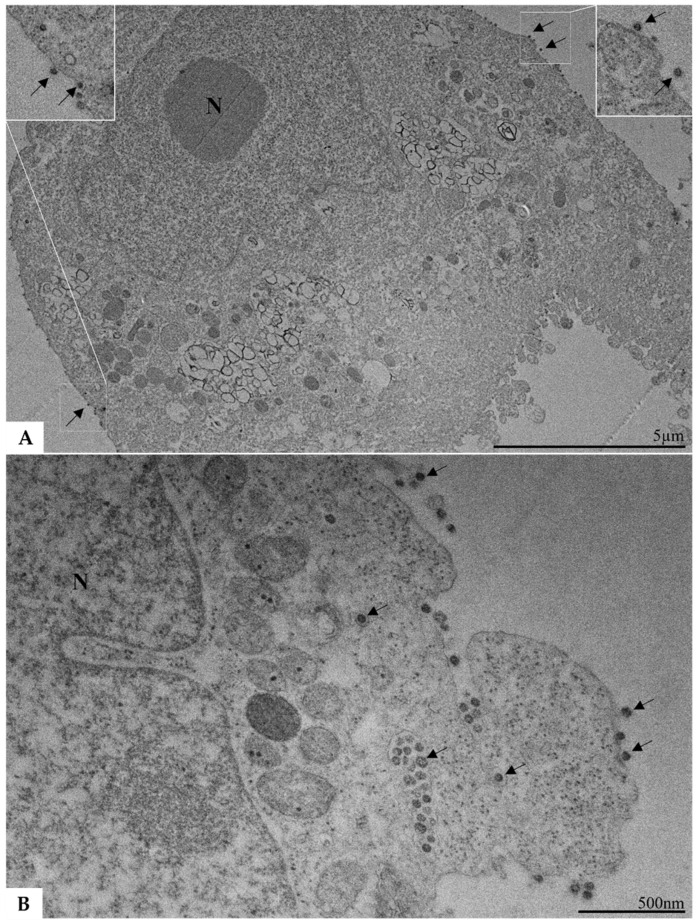
Ultrastructural analysis of cells infected with non-inactivated SARS-CoV-2 using transmission electron microscopy (**A**,**B**). Viral particles (indicated by arrows) can be observed to be attached to the cytoplasmic membrane (**A**,**B**) and within vesicles in the cell lumen (**B**).

**Figure 4:**
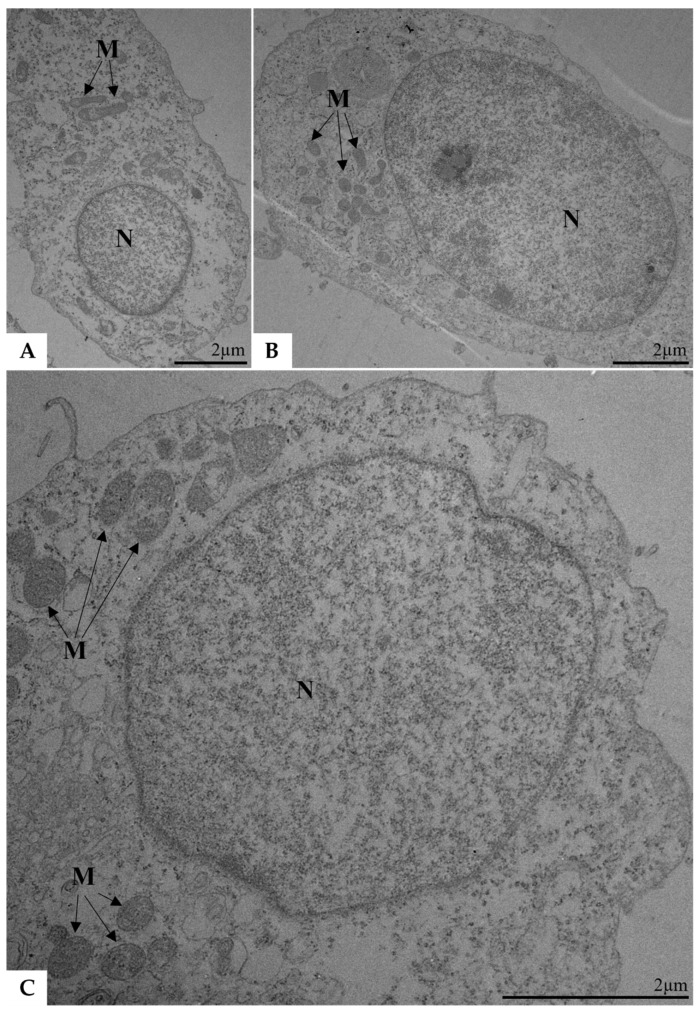
Vero E6 cells inoculated with inactivated SARS-CoV-2 and collected 48 h post-infection (**A**–**C**). Viral particles were not observed. N, nucleus; M, mitochondria. Transmission electron microscopy.

Vero cells infected with SARS-CoV-2, those exposed to inactivated SARS-CoV-2, and the negative control group were subjected to analysis using TEM at 48 pih as part of the replication kinetics experiment described earlier. Vesicles containing viral particles were observed within the cytosol in cells infected with SARS-CoV-2. Additionally, SARS-CoV-2 particles were detected attached to the cytoplasmic membrane (Figure 3A,B). In contrast, all cells exposed to inactivated SARS-CoV-2 (Figure 4) exhibited preserved structural integrity and the absence of viral particles, resembling uninfected cells.

## 3. Discussion

The SARS-CoV-2 pandemic posed challenges to health, politics, and society, prompting studies to understand the virus–host relationship and develop strategies like antivirals and vaccines to reduce spread. Diagnostic approaches, including RT-qPCR, have been used for early detection and isolation. Positive PCR results have helped populate databases, generate statistics, and prevent spread by isolating patients during the transmission period.

The RT-PCR method, recommended by the Centers for Disease Control and Prevention (CDC), confirms SARS-CoV-2’s presence via nucleocapsid targets [9]. However, positive RT-qPCR results do not always indicate viral infectivity [11], which can be assessed using nucleic acids from the viral replication cycle, such as subgenomic RNAs (sgRNAs) and intermediate RNAs [8]. These can help quantify the viral load in samples inactivated with agents like β-propiolactone for biotechnological purposes. Conventionally, the viral load of samples is evaluated using viral titration methods that measure the viral titer based on the mean infectious dose in tissue culture per mL (TCID50/mL) or a plaque assay (PFU/mL). The primary drawback of these methods is the extended time required to obtain results [10,12].

A study by Gomes et al. identified βPL inactivation as the most effective method for preserving viral particle morphology while inducing a robust immune response in murine models [10]. This approach holds significant potential for advancing immunobiological and technological projects. Traditionally, SARS-CoV-2 inactivation studies were limited to Biosafety Level 3 (BSL-3) laboratories due to stringent safety requirements [13,14]. However, updated WHO guidelines now permit the handling of SARS-CoV-2 diagnostic samples in BSL-2 laboratories, reducing reliance on high-containment facilities, which are often hindered by restricted access, high operational costs, and logistical challenges [15].

Despite these changes, ensuring viral inactivation and confirming non-viability remain essential in BSL-2 settings to safeguard personnel and ensure reliable experimental outcomes. Such validations involve detecting the absence of cytopathic effects in cell culture assays and verifying reduced viral loads through RT-qPCR analyses [8,10]. By incorporating these measures, researchers can confidently work in less restrictive environments while adhering to biosafety standards.

Using RT-qPCR for biotechnological product development enhances robustness and accuracy in quantifying viral load and monitoring inactivated viruses compared to cell-culture-titration methods. This technique was applied to quantify genomic RNA targets (N1, N2, and ENV), subgenomic RNAs (sgENV), and intermediate envelope RNAs (ENV-) to monitor viral replication and assess inactivation efficiency. The study confirms that RT-qPCR can detect and quantify genomic and subgenomic regions of SARS-CoV-2 RNA in inactivated virus samples. These findings align with the study by Vogels et al. on amplification efficiency and N and ENV viral load detection [16]. The oligonucleotide sequences for the nucleocapsid and envelope used in the study, which are based on those developed by Corman and Wölfel, demonstrated high specificity in detecting N and ENV [17].

This makes them promising targets for the confirmation of SARS-CoV-2 infection. The high specificity of these sequences not only ensures the reproducibility of results but also enhances the credibility and reliability of the technique. Consequently, this promotes the widespread adoption and dissemination of this methodology within the biotechnology field, contributing to advancements in diagnostic accuracy and research consistency.

Conducting studies under these revised protocols broadens the scope of biotechnological research. It not only minimizes the risk of live pathogens in lower-containment settings but also maintains the integrity of immunological and virological investigations. These advancements support innovation and progress in biotechnology, enabling safer and more efficient operations aligned with international safety regulations.

Our analysis revealed that βPL-inactivated samples exhibited reduced viral loads after blind serial passages, regardless of the variant, indicating impaired viral replication. This aligns with previous findings [10]. Detection of intermediate RNA and sgRNA confirms the non-replicative state of the inactivated virus. Current data indicate that sgRNA detection is more sensitive and specific for viable viruses than genomic RNA analysis [8]. Despite some conflicting studies [18], sgRNAs and other RNAs generated within double-membrane vesicles are exported and actively contribute to the formation of new particles [5,19,20]. This higher sensitivity, attributed to the detection of sgRNA and intermediate RNA, confirms the non-replicative state of inactivated viruses and serves as a sensitive marker of active viral replication, whereas genomic RNA can be present even in inactivated particles. By focusing on sgRNA, it is possible to differentiate between active and non-replicating viral particles, increasing the accuracy of viral viability assessments. Additionally, sgRNA detection via RT-qPCR serves as a specific measure of viral replication, particularly in preclinical vaccine development [7].

Data in the present study corroborate the work by Kim et al. [21], who stated that sgRNA detection via qPCR is a crucial factor in distinguishing viable from non-viable viral particles. It works as a replication and infection indicator. Unlike the aforementioned study, the present one was able to quantify sgRNA levels in viral cultures by using RT-qPCR, and it added greater robustness to the collected data, since the adopted technique showed high sensitivity and is considered the gold standard for nucleic acid quantification.

Amplification was not expected in inactivated samples targeting sgRNA, but the BS1 and BS2 passages showed quantification near 5 log10 copies/mL, likely due to insufficient purification. To address this, residual RNA degradation was performed using endonucleases common in immunobiological studies [22]. Oristo and collaborators demonstrated endonucleases’ effectiveness in degrading residual RNAs in rotavirus and norovirus samples, as well as reducing their levels via RT-qPCR [22].

Envelope sgRNA levels dropped to undetectable levels, highlighting RNase A’s efficiency [22]. Benzonase was not effective in reducing SARS-CoV-2 RNA levels, while 700 µg of RNase successfully reduced sgRNA in inactivated samples. It was not possible to compare the results of the aforementioned article with findings in the current study because the RNase A concentration used in the other article was not mentioned.

A kinetics assay was performed up to 48 pih to gather comprehensive data on SARS-CoV-2 replicative fitness. The findings align with Mautner et al., showing a rapid increase in RNA copies in the first 48 pih for various SARS-CoV-2 variants in Vero E6 cells [23]. Intermediate RNA and sgRNA detection provided insights into the viral replicative profile, showing high levels of all targets at 24 and 48 pih, particularly highlighting RNAs involved in replication (ENV and sgENV).

These RNAs, which arise during viral replication, serve as indicators of SARS-CoV-2 replication and active infection. The study’s results are consistent with the existing literature [24], showing significant sgRNA increases at 24 pih, corroborating gRNA level increases and supporting TEM analyses that highlighted exocytosis of new viral particles at 48 pih. Previous studies also showed RNA-like filaments in double-membrane vesicles at 48 pih [25], indicating these sites as RNA generation centers essential for producing structural proteins for new viral particles [26].

The replication kinetics assay clarified the timing of subgenomic RNA generation, which actively participates in synthesizing SARS-CoV-2 structural proteins. This correlates with TEM data, showing new viral particles undergoing exocytosis at 48 pih. Monitoring was conducted up to 48 pih; a longer period was not observed due to limited access to BSL-3 facilities. Extending this period would help identify the viral replication peak and improve our understanding of RNA detection limits in viral protein synthesis.

Our study of viral inactivation, including βPL inactivation, improves vaccine development and diagnostics by providing precise viral viability assessments. Utilizing RT-qPCR to detect subgenomic RNAs (sgRNAs) ensures higher sensitivity and specificity in identifying active viral replication, which is essential for evaluating the effectiveness of inactivated vaccines. This advancement facilitates immunobiological and diagnostic research, thereby accelerating the creation of vaccines and diagnostic tools. By connecting our findings to practical applications, the study underscores its importance for public health.

Despite certain methodological limitations, we highlight the significance of sgRNA and intermediate RNA detection in confirming SARS-CoV-2 replication and supporting the use of inactivated viruses in immunobiological development. This approach allows for accurate monitoring of viral activity, enhancing the safety and effectiveness of biotechnological applications and significantly advancing virological research and public health initiatives.

## 4. Materials and Methods

SARS-CoV-2 cultures, as well as infectious SARS-CoV-2 procedures, were conducted in a Biosafety Level 3 (BSL-3) facility in compliance with approved international laboratory biosafety guidelines [9,13].

### 4.1. Cell Cultivation

SARS-CoV-2 was cultured in Vero E6 cells derived from the kidney of an African green monkey (*Cercopithecus aethiops*, ATCC, CRL-1586, Manassas, VA, USA). Cells were cultured in Earle’s 199 medium with salts, supplemented with 5% Fetal Bovine Serum (FBS), 40 μg/mL gentamicin sulfate, and 2 mM L-glutamine (Gibco, Waltham, MA, USA; Sigma-Aldrich, Burlington, MA, USA). The cell line was maintained at 37 °C under 5% CO_2_.

### 4.2. Viral Production and Inactivation

Viral stocks in BLS-3 facilities were produced with 0.01 multiplicity of infection (MOI) in stationary cultures of Vero E6 cells, with a cell concentration of 70,000 cells/cm^2^ prepared 24 h prior to viral infection in a 175 cm^2^ flask. The inoculum was removed after the adsorption step, which lasted for 1 h at 37 °C; then, the cells were maintained in either 199 media supplemented with 2% FBS and 2 mM L-glutamine or OptiPRO medium (Gibco, Waltham, MA, USA) supplemented with 4 mM L-glutamine. Infected cells were incubated for 2 days at 37 °C under 5% CO_2_. The supernatant was collected after the incubation period, and the viral suspension was clarified in a sterilizing filtration system with a 0.22 µm pore size (Merck, Darmstadt, Hesse, Germany). Subsequently, 8% *w*/*v* D-Sorbitol (Sigma-Aldrich, Burlington, MA, USA) was added to the supernatant to ensure viral stability at low temperatures. Viral stocks were stored at −80 °C until use (Appendix A, Table A1). Viral stocks were subjected to the chemical agent βPL (©Natalex, Warsaw, Mazowieckie, Poland) at a dilution of 1:3000 (0.03%) for 24 h at 4 °C, based on a previously described protocol [10]. βPL induces viral inactivation through viral RNA alkylation based on the addition of alkyl groups to the viral genomic material to render it incapable of transcription [14,27]. The viral samples used in these assays belonged to SARS-CoV-2 Wuhan.

### 4.3. Blind Serial Passage

The blind serial passage methodology was employed in Vero E6 cells to confirm viral inactivation by βPL. It consisted of five consecutive passages of SARS-CoV-2 inactivated with βPL, as previously described [10]. Vero E6 cells were prepared in T-25 cm^2^ flasks, at a cell concentration of 100,000 cells/cm^2^, 24 h before viral infection. Likewise, flasks were prepared to assess the controls, including the non-inactivated virus (positive control). BSL-3 cells were inoculated with 500 µL of viral sample and incubated at 37 °C under 5% CO_2_ for 3 days. Subsequently, a new flask filled with 100,000 cells/cm^2^ and 10 mL of medium was inoculated with 500 µL of supernatant from the previous flask. New passages were carried out after the incubation period by following this same protocol, and it totaled five passages. Aliquots of inactivated samples and their corresponding controls were collected at each successive passage. These samples were used for viral load analysis via RT-qPCR amplification assays targeting nucleocapsids (N1 and N2) and the SARS-CoV-2 envelope gene (ENV).

### 4.4. Replication Kinetics

Vero E6 cells were seeded in six-well plates at a cell concentration of 60,000 cells/cm^2^, supplemented with Earle’s 199 medium with 2% FBS and 2 mM L-glutamine, 24 h before the assay to assess the SARS-CoV-2 replicative profile. Plates were inoculated with both active SARS-CoV-2 (positive control) and inactivated SARS-CoV-2, as pre-established, considering the cell density, well area, and 0.01 MOI [10]. The cell monolayer was washed twice in 3 mL of PBS 1×, at pH 7.4, following the adsorption step; then, 3 mL of Earle’s 199 medium supplemented with 2% FBS was added. The plates were incubated for 1, 2, 3, 24, and 48 post-infection hours (pih). Aliquots of 140 µL of the supernatant from each well, including the controls, were transferred to microtubes filled with 560 µL of lysis buffer (AVL) from a QIAmp Viral RNA Mini Kit (QIAGEN^®^, Hilden, North Rhine-Westphalia, Germany) for RT-qPCR analysis purposes at the end of each incubation. Subsequently, the cell monolayer was washed 3 times in 1 mL of PBS 1×, at pH 7.4, in order to remove supernatant remnants. An aliquot of 140 µL of PBS 1×, at pH 7.4, and 560 µL of AVL were added to the cell monolayer. Approximately 700 µL of cell lysate was collected and transferred to pre-identified microtubes. Cell control was achieved by segregating a non-inoculated plate kept without BSL-3 manipulation.

### 4.5. Transmission Electron Microscopy (TEM)

The supernatants from three wells at the 48 pih time point were discarded to assess the viral infection’s effect on Vero E6 cells. An aliquot of 500 µL of trypsin (Gibco, Waltham, MA, USA) was added to the cell monolayer and maintained for 2 min. Subsequently, 500 µL of FBS was added to neutralize the trypsin’s action. The cell suspension was collected from all wells and combined into a single tube to form the cell pool, which was transferred to a conical tube. Subsequently, cells were fixed in 1% glutaraldehyde in 0.1 M sodium cacodylate buffer, at pH 7.2. Samples were washed in 0.1 M sodium cacodylate buffer with 7% sucrose, post-fixed in 1% osmium tetroxide for 4 h at 4 °C, and dehydrated in increasing acetone bath concentrations after they were fixed. Then, cells were infiltrated with epoxy resin and kept in an oven at 60 °C, for 3 days, for polymerization purposes [27,28]. Ultrathin sections (30–60 nm) were obtained using a diamond knife (DiATOME, Nidau, Bern, Switzerland) coupled to the ultramicrotome (Reichert-Jung, Wetzlar, Hesse, Germany), collected on 300-mesh copper grids (Electron Microscopy Sciences, Hatfield, PA, USA), and analyzed on the Hitachi HT7800 transmission electron microscope (Hitachi, Tokyo, Japan) of the Rudolf Barth Electron Microscopy Platform, Oswaldo Cruz Institute.

### 4.6. Viral RNA Extraction

RNA extraction was performed in a QIAamp Viral RNA Mini Kit (QIAGEN^®^, Hilden, North Rhine-Westphalia, Germany) according to the manufacturer’s recommendations. This methodology is based on subjecting 140 µL of each sample to cellular lysis carried out through the action of denaturing agents that act to inactivate the RNases found in the viral supernatant. From this step onwards, RNA is expected to bind to a silica gel column; then, successive washing and elution steps are carried out. The eluted volume of 60 µL of extracted material was stored at −80 °C until use.

### 4.7. Reverse Transcription Followed by SARS-CoV-2 Genomic RNA Quantitative Polymerase Chain Reaction (RT-qPCR)

Genomic RNA (gRNA) quantification was performed using three targets: two nucleocapsid genes (N1 and N2) and the SARS-CoV-2 envelope gene (ENV). The 2019-nCoV CDC RUO kit (Integrated DNA Technologies—IDT, Coralville, IA, USA) was used, which is the reference kit for SARS-CoV-2 detection from the Centers for Disease Control and Prevention (CDC) in the United States [9]. Oligonucleotides described by Corman et al., 2020 [17] were used for ENV target analysis (Table 1).

Positive polarity (gRNA) nucleic acid quantification was performed in a TaqManTM Fast Virus One-Step Master Mix Kit (Applied Biosystems, Foster City, CA, USA) to enable reverse transcription (RT) and qPCR amplification in a single reaction. The amplification reaction of N1 or N2 targets consisted of 1.4 µL of kit mix (2019-nCoV CDC RUO) added to sense and antisense primer oligonucleotides (500 nM/reaction) and 0.5 µL of probe (250 nM/reaction) supplemented with 8.6 µL of DNase and RNase-free water, 5.0 µL of TaqMan^®^ Fast Virus Master, and 5.0 µL of sample RNA, which totaled 20 µL per reaction for each independent target. The reaction mix for the ENV target was prepared with a total volume of 20 µL. It was composed of 1 µL of each sense and antisense primer oligonucleotide (500 nM/reaction), 0.5 µL of probe (250 nM/reaction), 7.5 µL of DNase and RNAse-free water, 5.0 µL of TaqMan^®^ Fast Virus Master, and 5.0 µL of sample RNA. Reagents were handled under aseptic conditions, and 15 µL from the mix was applied to a 96-well RT-qPCR plate (Applied Biosystems, Foster City, CA, USA). Genetic material was added to the plate containing the mix; then, the plate was sealed, centrifuged, and inserted into the 7500 System for Real-Time PCR (Thermo Fisher Scientific, Waltham, MA, USA) to start the reaction in another aseptic chamber.

### 4.8. Reverse Transcription Followed by Quantitative Polymerase Chain Reaction (RT-qPCR) of Subgenomic RNA from the SARS-CoV-2 Envelope Region

The sgRNA’s presence was assessed by detecting the sgENV envelope region, which is one of the most expressed genes during the SARS-CoV-2 virus replicative cycle [7]. Oligonucleotides described by Wölfel et al., 2020 [8] were employed in order to do so (Table 1). The reaction mix consisted of 1 μL of each sense and antisense primer oligonucleotide (500 nM/reaction), 0.5 μL of probe (250 nM/reaction), 7.5 μL of DNase and RNase-free water, 5 μL of TaqMan^®^ Fast Virus Master, and 5 μL of RNA from the samples, which totaled 20 μL per reaction.

### 4.9. Reverse Transcription Followed by Quantitative Polymerase Chain Reaction (RT-qPCR) of Negative-Polarity RNA from the SARS-CoV-2 Envelope Target

Reverse transcription of the negative strand was performed by using a sense oligonucleotide for the envelope region (ENV) to amplify and quantify the negative-polarity intermediate RNA involved in the SARS-CoV-2 replication cycle (Table 1), and it led to complementary DNA (cDNA) generation, which was subsequently amplified by qPCR. The High-Capacity cDNA Reverse Transcription Kit with RNase Inhibitor (Applied Biosystems, Foster City, CA, USA) was used for cDNA synthesis purposes. The reaction mix was prepared to a final volume of 20 µL, and it consisted of 3.5 µL of DNase and RNase-free water, 2 µL of forward oligonucleotide, 2 µL of reaction buffer, 1 µL of dNTPs, 0.5 µL of RNase Inhibitor, 1 µL of multiscribe, and 10 µL of genetic material. An aliquot of 5 µL of cDNA was used in qPCR by employing the same protocol described for the envelope region to amplify, detect, and quantify negative strands.

### 4.10. Standard Curve Construction

Plasmids containing nucleocapsid (N1/N2), envelope (ENV), and SARS-CoV-2 subgenomic envelope (sgENV) genes were obtained through Integrated DNA Technologies (IDT, Coralville, IA, USA)—a commercial supplier—to generate the standard curves of the targets to be used. Target N1/N2 genomic sequences with 349 base pairs (bp), the ENV target with 315 bp, and the sgENV target with 181 bp were derived from the Wuhan variant. They were inserted into the commercial plasmids CAT_10006625_2019-nCoV_N_Positive Control, CAT_10006896_2019-nCoV_E Positive Control, and pIDTSMART-AMP.

### 4.11. Results Analysis

Assay results were analyzed in ABI 7500 instrument software (Thermo Fisher Scientific, Waltham, MA, USA), based on the logarithmic profile observed in the amplification and multicomponent graphs, in order to assess whether there was amplification higher than the baseline (0.03). The amplification of each sample resulted from the number of thermal cycles (Ct) required for fluorescence detection by the equipment. The Ct was subjected to linear regression applied to the standard curve of each specific target based on this value; then, the number of copies/mL was calculated. Given the limited sample size, the nonparametric Mann–Whitney test was employed to validate the data, ensuring that the observed differences between conditions were statistically significant and not attributable to chance, thereby enhancing the accuracy and reliability of the results. GraphPad Prism 8 (Version 8.0.1,Boston, MA, USA) software was used to plot the graphs.

## 5. Conclusions

Although we are in post-pandemic times and the biosafety guidelines for SARS-CoV-2 have been updated, its handling still requires caution. Data reported in this study contribute to the monitoring of SARS-CoV-2 viral inactivation and its safe use in biotechnological processes. The evaluation of blind serial passages using the RT-qPCR method with genomic and subgenomic targets provides strong evidence that this sensitive, specific, and rapid method can be an excellent alternative for assessing viral replication in the pharmaceutical industry.

## Figures and Tables

**Table 1:** Oligonucleotides used in RT-qPCR for SARS-CoV-2 envelope (ENV) and nucleocapsid (N1 and N2) targets.

Gene	Oligonucleotide	Sense	Sequence 5′-3′	Genome Position
Envelope (ENV)	E_Sarbeco_F	Forward	ACAGGTACGTTAATAGTTAATAGCGT	26269–26294
	E_Sarbeco_R	Reverse	ATATTGCAGCAGTACGCACACA	26360–26381
	E_Sarbeco_P1	Probe	FAM-ACACTAGCCATCCTTACTGCGCTTCG-BHQ1	26332–26357
Subgenomic envelope (sgENV)	sgLeadSARSCoV2-F	Forward	CGATCTCTTGTAGATCTGTTCTC	44–66
	E_Sarbeco_R	Reverse	ATATTGCAGCAGTACGCACACA	26360–26381
	E_Sarbeco_P1	Probe	VIC-ACACTAGCCATCCTTACTGCGCTTCG-MGBNFQ	26332–26357
Nucleocapsid (N)	2019-nCoV_N1-F	Forward	GACCCCAAAATCAGCGAAAT	28287–20306
	2019-nCoV_N1-R	Reverse	TCTGGTTACTGCCAGTTGAATCTG	28335–28358
	2019-nCoV_N1-P	Probe	FAM-ACCCCGCATTACGTTTGGTGGACC-BHQ1	28309–28332
	2019-nCoV_N2-F	Forward	TTACAAACATTGGCCGCAAA	29164–29183
	2019-nCoV_N2-R	Reverse	GCGCGACATTCCGAAGAA	29213–29230
	2019-nCoV_N2-P	Probe	FAM-ACAATTTGCCCCCAGCGCTTCAG-BHQ1	29188–29210

REF: hCoV-19/Wuhan/WIV04/2019 (WIV04); https://gisaid.org/wiv04/ (Accessed on 15 November 2024).

## Data Availability

Data introduced in the current study are available upon request to the corresponding author.

## Appendix A

**Table A1:** SARS-CoV-2 Strains Used in the Study.

Strain	Variant	State	Municipality	GISAID ID *
Gama	P1	AM	Manaus	EPI_ISL_1402431
Alfa	B1.1.7	RJ	Rio de Janeiro	EPI_ISL_1402430
Zeta	P2	AL	Maceió	EPI_ISL_792642
Whuan	-	RJ	Rio de Janeiro	EPI_ISL_414045
Ômicron	BA.1	SC	Jaraguá do Sul	EPI_ISL_8430488

* GISAID {XE “GISAID”\t “*Global Initiative on Sharing All Influenza Data*”}, Iniciativa de Compartilhamento de Dados (https://gisaid.org/, Accessed on 15 November 2024).
